# Proceedings of Pain Science in Motion Colloquium—3rd edition. May 31st—June 2nd, University of Genoa-Campus of Savona, Italy: **Giving insight in pain research of tomorrow!**

**DOI:** 10.1097/PR9.0000000000000753

**Published:** 2019-05-25

**Authors:** C. Paul van Wilgen, Marco Testa

The research findings and the pain models of tomorrow can be found in the research questions of today. Therefore, 7 years ago, within the Pain in Motion group, the idea was launched to start a podium dedicated to PhD researchers. In contrast to traditional congresses, the idea was to present starting or ongoing research. This resulted in the first Pain Science in Motion Colloquium. Researchers were invited not to present existing data and finished research, but primarily to present starting research projects with their underlying theories and designs. This gave the chance for young researchers to present on an international stage early in their career, to meet fellow PhD pain researchers and discuss and share their research. Moreover, young researchers have the opportunity to encounter 5 senior researchers that are invited to give a keynote lecture in the Pain Science in Motion Congress, but in particular to discuss with them during the “meet the expert” sessions.

After Brussels (2015) and Stockholm (2017), the *III edition of the Pain Science in Motion colloquium* will be held in 2019 in Savona, Italy, at the Campus of the University of Genoa. This year, thanks to a multidisciplinary group of PhD researchers coming from all over the world, the program offers 8 oral sessions with 40 presentations and 10 thematic poster sessions with 50 posters with short interactive presentations.

The keynote experts invited will be Prof. Fabrizio Benedetti (University of Torino, Italy) Prof. Rob Smeets (University of Maastricht, Netherlands), Jessica van Oosterwijck (University of Gent/Antwerpen, Belgium), Prof. Alberto Gallace (University of Milano Bicocca, Italy), Prof. Deborah Falla (University of Birmingham, UK).

We hope that the reading of the short abstracts of the selected oral presentations can be inspiring for future young researchers who will increase the quality of the forthcoming editions of the Pain Science in Motion Colloquium.

More information about the present colloquium, the 2019 version and future editions can be found at the congress website: www.PSIM2019.org or at the website of Pain in Motion: http://www.paininmotion.be

On behalf of the Scientific Committee

Prof Dr. C. Paul van Wilgen

VU Brussels, Transcare pain (www.transcare.nl) Pain in Motion

Chair of the Scientific Committee

Prof Dr. Marco Testa

Department of Neuroscience, Rehabilitation, Ophthalmology, Genetics, Maternal and Child Health, University of Genova—Campus of Savona

Chair of the Organizing Committee

Member of the Scientific Committee

## Offset analgesia in patients with migraine and healthy controls

Tibor Szikszay^a^, Waclaw Adamczyk^a,b^, Kerstin Luedtke^a,c^

^*a*^*Pain and Exercise Research Luebeck (P.E.R.L), University of Luebeck, Luebeck, Germany,* ^*b*^*Department of Kinesiotherapy and Special Methods in Physiotherapy, The Jerzy Kukuczka Academy of Physical Education, Katowice, Poland,* ^*c*^*The Jerzy Kukuczka Academy of Physical Education, Katowice, Poland*

Migraine is a common and debilitating disease, but the pathophysiology is poorly understood. Dysfunctional endogenous pain modulation is discussed as a contributing factor to the development and/or maintenance of the disease. Offset analgesia (OA) is a frequently used paradigm to identify endogenous pain modulation. The aim of this study is to assess OA in patients with migraine and healthy controls. Twenty-one patients with migraine and 21 healthy age and gender matched healthy controls were recruited. In both groups, selected tests from the quantitative sensory testing protocol were assessed. OA was performed using a three-temperature stimulus paradigm on both sides of the forehead and the forearm. An individualized temperature of 50/100 for 5 seconds (T1), +1°C for 5 seconds (T2), and again the individualized temperature for 20 seconds (T3) were applied. In addition, 3 constant temperature stimuli of T1 were applied for 30 seconds. The constant and offset trials as well as the examined body regions were performed in a randomized order. Results and conclusions: The project is in its final phase. To date, 15 patients with migraine (examined interictally) and 15 healthy controls have been included.

## A Delphi study to identify care priorities for South African patients suffering with phantom limb pain after limb amputations

Katleho Limakatso^a^, Victoria J. Madden^b^, Romy Parker^b^

^*a*^*Pain Management Unit, Department of Anaesthesia and Perioperative Medicine, Faculty of Health Sciences, University of Cape Town, Cape Town, South Africa,* ^*b*^*University of Cape Town*

A patient-led Delphi study will be conducted using a convenience sample of patients who have undergone limb amputations and are suffering from phantom limb pain (PLP). This study will undergo 3 sequential rounds of anonymous online questionnaires, following which a consensus will be reached on care priorities for people with limb amputations. Thereafter, a subsample of participants will be chosen randomly and interviewed to provide a rationale for their ranking. Data collected from the interviews will be analysed qualitatively using an open coding process. The rest of the data will be analysed by calculating measures of central tendency (median and interquartile ranges). A consensus will be reached when the interquartile range is ≤1, indicating an agreement of more than 50%. This proposed study is currently in development. Ethical approval to conduct this study will be sought from the relevant Ethics Committee at the University of Cape Town. According to our knowledge, this will be the first ever Patient-led Delphi study to identify care priorities for patients who have undergone limb amputations and seek treatment for PLP. The results of this study are expected to contribute towards the effective management of people with amputations, and direct future research on this subject.

## Self-reinforcement mechanism of the placebo effect in painful paraesthesia

Waclaw Adamczyk^a^, Andrzej Bieniek^b^, Piotr Wodarski^b^, Aleksandra Budzisz^c^, Tibor Szikszay^a^, Kerstin Luedtke^a^

^*a*^*Pain & Exercise Research Luebeck, University of Luebeck, Luebeck, Germany,* ^*b*^*Department of Biomechatronics, Silesian University of Technology, Zabrze, Poland,* ^*c*^*Department of Pedagogy and Psychology, The Jerzy Kukuczka Academy of Physical Education, Katowice, Poland*

The aim of this project is to explore the response-generalization of the placebo effect in a novel paradigm which induces paraesthesia and pain by transient limb ischemia. Placebo effect will be induced by classical conditioning with self-reinforcement procedure. Healthy volunteers will be exposed to series of ischemic stimuli delivered by an electronic sphygmomanometer to their non-dominant arm. Pain and paraesthesia will be measured continuously by 2 computerized scales. Physiological correlates of bodily symptoms will be measured by skin conductance. After the pretest phase, subjects from group 1 will receive a placebo cream together with verbal suggestions on its effectiveness (tingling reduction). Then, they will undergo a conditioning procedure (surreptitious increase of pressure). In group 2, a conditioning will include a self-reinforcement in which pressure will be surreptitiously increased gradually. In group 3 verbal suggestion alone will be used and in group 4 no intervention takes place (control). In pilot study, we surprisingly found that higher pressure paradoxically causes less paraesthetic symptoms and lower pressure induces paraesthesia of higher intensities. The study is elucidating a new mechanism by which the placebo effect in paraesthesia might by induced, a self-reinforcement mechanism.

## Observing transdisciplinary pain neuroscience education practice: what kind of processes are involved?

Amarins Wijma^a,b,c^, Tineke Pieterson^d^, Lennard Voogt^c,e^, Doeke Keizer^b^, Jo Nijs^c,f^, Paul. C. van Wilgen^b,c,g^

^*a*^*Faculty of Physical Education and Physiotherapy, Vrije Universiteit Brussel, Brussels, Belgium,* ^*b*^*Transcare Transdisciplinary Painmanagement Center, The Netherlands,* ^*c*^*Pain in Motion research group,* *www.paininmotion.be**,* ^*d*^*Fysiotherapie Jissink, Sneek,* ^*e*^*Department of Physical Therapy, Rotterdam University of Applied Sciences, The Netherlands,* ^*f*^*Department of Physiotherapy, Human Physiology and Anatomy, Faculty of Physical Education and Physiotherapy, Brussels, Belgium,* ^*g*^*Department of Physiotherapy and Rehabilitation Sciences, Faculty of Physical Education & Physiotherapy, Vrije Universiteit Brussel, Brussels, Belgium*

There has, to our knowledge, not yet been a study investigating the interaction between the patient and healthcare professional(s) during pain neuroscience education (PNE). As PNE is a “talk-modality” many interpersonal aspects contribute to the outcome of the treatment. Therefore, the purpose of this study was to investigate the interaction between the patient and healthcare professionals and influence of these interactions on the outcome of the PNE. Even more specific: to understand and comprehend if and when patients with chronic pain accept PNE and which processes lead to this acceptance. Based on Constructive Grounded Theory (CGT) a heterogenic convenience sample 8 respondents were recruited from a transdisciplinary outpatient center between March 2015 and December 2016. We conducted an observational qualitative study to grasp and theorize the practice of PNE. Nine unobtrusive video observations of the interactions of a physiotherapist, psychologist and patients with chronic pain during the PNE in a transdisciplinary setting were made. The observations are currently analyzed according to CGT. Quality of the study is assured by following the trustworthiness criteria of Licoln and Guba. The outcome of the study will be a theoretical framework and construct. Results: Not yet available. Conclusions: Observing practice according to CGT is not often performed within chronic pain practice. Within the field of PNE this is to our knowledge the first study. Nonetheless, understanding how healthcare professionals implement and integrate evidence-based practices such as PNE is crucial for the further development of the therapy.

## Association between central sensitization and lifting and aerobic capacity in patients with chronic low back pain

Jone Ansuategui Echeita, Rienk Dekker, Henrica R. Schiphorst Preuper, Michiel F. Reneman

Department of Rehabilitation Medicine, University of Groningen, University Medical Center Groningen, Groningen, The Netherlands

**Introduction:** Analyze the relationship between Central Sensitization (CS), and lifting and aerobic capacities in patients with Chronic Low Back Pain (CLBP). Describe pain response to strenuous exercise in patients with CLBP. Observational longitudinal with measurements at baseline and discharge.

**Measurements:** CS with Central Sensitization Inventory (CSI), Lift capacity with floor-to-waist lifting test, Aerobic capacity with Cardiopulmonary Exercise Test (CPET), and Pain response with Pain response questionnaire.

**Statistical analyses:** Stepwise-forward regression: change between baseline and discharge of lift and aerobic capacities (dependent), and of CSI (independent). Repeated measures ANOVA: pain intensity before CPET compared to immediately and 24 hours after; at baseline and discharge.

**Results (preliminary):** Data collection is ongoing; currently data of 11 patients are collected. A trend of decreased CSI associated with increased performance is seen in lifting but not in aerobic capacity. Immediate pain responses at intake and discharge show decreasing trend in low back while significantly increasing in legs.

**Conclusions (preliminary):** Patients with CLBP may show some influence of CS level on their lifting capacity. Following strenuous exercise leg pain may increase as opposed to low back pain; diffuse noxious inhibitory control could possibly explain the effect.

## What is the influence of low back pain on muscle activity and movement during a cyclical dynamic task?

Andy Sanderson^a^, Corrado Cescon^b^, Nicola Heneghan^a^, Alison Rushton^a^, Marco Barbero^b^, Deborah Falla^a^

^*a*^*CPR Spine, University of Birmingham,* ^*b*^*University of Applied Sciences and Arts of Southern Switzerland, SUPSI*

**Introduction:** Low back pain (LBP) is a leading cause of disability. High-density electromyography (HDEMG) has revealed differences in the spatial distribution of back muscle activity during movements. However, these studies have only considered a small portion of the erector spinae (ES) during tasks which were either static or mono-planar.

**Methods:** This study combines HDEMG and kinematic analysis to investigate the effect of LBP on the spatial distribution of ES activity during a dynamic lifting task. Sixteen people with chronic LBP (8 male, age: 26.9 ± 10.8 years) and 16 age and gender-matched controls (7 male, age: 31.7 ± 14.0 years) completed the study. HDEMG signals from the ES were detected bilaterally by four 64-channel semi-disposable 13 × 5 electrode grids. Kinematic surface markers were placed over the back in triangular arrangements, creating lumbar and thoracic segments to track movement. HDEMG and kinematic data were recorded continuously during a dynamic task involving the cyclical lifting of a 5 kg box between 6 shelves for 10 cycles (∼7 minutes). The shelves were arranged around the participant, at knee and sternal height.

**Results:** Data analysis is underway; with full results presented at the conference.

**Conclusions:** This study will impact on our understanding of neuromuscular adaptations to LBP during functional activity.

## Comparison of the impact of central sensitization between patients with chronic low back pain and knee osteoarthritis

Katsuyoshi Tanaka^a,b^, Tomohiko Nishigami^c^, Akira Mibu^c^, Masahiro Manfuku^d^, Satoko Yono^a^

^*a*^*Department of Rehabilitation, Tanabe Orthopaedics, Osaka, Osaka, Japan,* ^*b*^*Department of Community Health Sciences, Kobe University Graduate School of Health Science, Kobe, Hyogo, Japan,* ^*c*^*Department of Nursing and Physical Therapy, Konan Women's University, Kobe, Hyogo, Japan,* ^*d*^*Breast Care Sensyu Clinic, Kishiwada, Osaka, Japan*

The aim of the present study was to investigate a difference in the impact of CS between types of disorders, patients with chronic low back pain (CLBP) and knee osteoarthritis (KOA). Patients with CLBP (n = 104) and KOA (n = 50) completed the Central Sensitization Inventory (CSI), the Brief Pain Inventory (BPI), EuroQoL 5-dimension (EQ5D), and the Pain Catastrophizing Scale (PCS). Correlations between the CSI, BPI, EQ5D, and PCS were investigated. We used the receiver operating characteristic curve for the CSI score to calculate the area under the curve (AUC) and cutoff points in order to discriminate pain interference in each group. The CSI was significantly higher for the CLBP group than for the KOA group (25.5 ± 12.2 and 17.6 ± 10.3). The CSI was significantly correlated with pain intensity, pain interference, EQ5D, and PCS for both groups. The AUCs were 0.73 for CLBP and 0.75 for KOA. Cutoff points of the CSI score were 34 for the CLBP and 18 for the KOA. There was a difference with cutoff point of the CSI score between the groups. We should consider the difference with cutoff point depends on patient group.

## Effectiveness of pain neuroscience education and physiotherapy in subjects scheduled for a total knee arthroplasty: randomized clinical trial

Marc Terradas-Monllor^a^, Mirari Ochandorena-Acha^a^, Jose Antonio Hernandez-Hermoso^b^, Héctor Beltran-Alacreu^c^

^*a*^*Research Group on Methodology, Methods, Models and Outcomes of Health and Social Sciences (M3O), Faculty of Health Sciences and Welfare, University of Vic, Central University of Catalonia, Vic, Barcelona, Spain,* ^*b*^*Orthopedic Surgery Service, University Hospital Germans Trias i Pujol, Badalona, Barcelona, Spain,* ^*c*^*Motion in Brains Research Group, Institute of Neuroscience and Movement Sciences (INCIMOV), Centro Superior de Estudios Universitarios La Salle, Universidad Autonóma de Madrid, Spain*

**Objectives:** The primary aim of this study is to investigate the effectiveness of 2 different preoperative physiotherapy interventions in patients scheduled for a TKA with pain catastrophizing and moderate to severe knee pain.

**Methods:** This study is a 3-arm parallel group trial design including 45 subjects with high levels of pain catastrophizing and moderate to severe pain, scheduled for total knee arthroplasty due to primary osteoarthritis. Patients eligible for participation will be randomized in 3 arms, usual care, usual care and pain neuroscience education, or usual care and multimodal physiotherapy. Measurements will be taken 4 months and 2 weeks before surgery, and 3 and 6 months after surgery. Primary outcome will be pain measured with VAS (Visual Analogue Scale), whereas secondary outcomes include physical function and psychosocial factors such as Kinesiophobia, quality of life and self-management.

**Discussion:** There are no studies that have investigated the effectiveness of a physiotherapy intervention on patients with preoperative risk factors before a TKA. This trial will provide new evidence regarding the existing health care recommendations on patients scheduled for a TKA.

## Investigating the repeatability and stability of exercise induced hypoalgesia in healthy adults

Pauline Kuithan, Alison Rushton, Nicola R. Heneghan, Deborah Falla

CPR Spine, University of Birmingham

No previous study has evaluated the reproducibility of exercise induced hypoalgesia over multiple sessions with the same intervention or its extent with repeated exposure to the same exercise. The aim of this study is to investigate the reliability and stability of the effects of EIH with a repeated endurance or a resistance exercise protocol. Healthy participants (18–55 years) will be randomised to 1 of 2 exercise interventions; either a brisk treadmill walking task or an isotonic lifting task, which will be repeated 6 times over 3 weeks. To assess EIH pre- and post-intervention, extensive quantitative sensory testing will be conducted over the back and remote body regions. Intra-class correlation coefficients for intra-rater reliability, Bland Altman plots for limits of agreement, and an analysis of variance using partial eta squared for effect size will be calculated. Preliminary results will be presented, as data collection is under way. Stability and reliability of results will further inform clinical decision making and elucidate its impact on patient outcomes to exercise. This study will be important for future considerations and research in people with musculoskeletal disorders to examine how EIH is modified in people with chronic pain.

## Central pain processing, psychosocial and lifestyle factors as potential moderators for outcome after rotator cuff repair: a procotol

Ariane Schwank^a,b^, Filip Struyf^b^, David Gisi^a^, Markus Pisan^a^, Mira Meeus^b^

^*a*^*Kantonsspital Winterthur,* ^*b*^*University of Antwerp*

**Introduction:** To explore the interrelationship between central pain processing (CPP) and outcome after rotator cuff repair (RCR) and to investigate whether modifiable psychosocial and lifestyle factors additionally influence the pain level, shoulder function and quality of life after RCR. The purpose is to identify moderators for outcome after RCR.

**Methods:** This longitudinal cohort study will investigate 141 standard care datasets of adult patients undergoing RCR at Kantonsspital Winterthur, Switzerland. Mean change of 3 measurement points, (1) pre-operative, (2) 12 weeks post-operative, (3) 12 months post-operative of primary (Western Ontario Rotator Cuff Index [WORC]) and secondary outcome measures (Constant—Score, maximum pain and quality of life) will be analysed by mixed-effects regression models for repeated measures. A stepwise inclusion of the 6 potential moderators will be conducted using linear and logistic regression models. Potential moderators are obtained by quantitative sensory testing and central sensitisation inventory (CPP), pain catastrophising scale, illness perception questionnaire, perceived stress scale and questions about expectations and sleep. Estimated in 2020.

**Conclusions:** Results may disclose moderators for outcome after RCR and foster a more disease specific knowledge, which supports an individualized approach towards the course of care and may affect the recovery rate for shoulder pain.

## The influence of physical activity on the nociceptive flexion reflex in healthy adults: a cross-sectional study

Evy Dhondt, Lieven Danneels, Sophie Van Oosterwijck, Tanneke Palmans, Johan Rijckaert, Jessica Van Oosterwijck

Ghent University

To investigate whether the nociceptive flexion reflex (NFR) threshold can be predicted by self-reported and objective measures of physical activity (PA). PA levels and NFR thresholds of 58 healthy adults were cross-sectionally determined. PA was evaluated during 7 consecutive days by self-report using the International Physical Activity Questionnaire, and by objective accelerometry data obtained using an activity monitor. The NFR was quantified by a biceps femoris muscle electromyogram following transcutaneous electrical stimulation of the sural nerve. Hierarchical linear regression analyses were performed to determine the relationship between PA and the NFR. Results indicate that individuals who perform more daily step counts show higher NFR thresholds. Furthermore, those who expend more energy and time on activities of moderate intensity level exhibit higher NFR thresholds. The current study provides preliminary evidence indicating that a physically active lifestyle may be beneficial for spinal nociception in healthy adults. These findings help improve our understanding on the endogenous effects of exercise. Given their potential to reduce spinal nociception, walking and moderate-intensity activities could be useful in the treatment program of chronic pain patients in which increased spinal nociception has already been demonstrated. Future research examining the latter hypothesis is warranted.

## The effectiveness of pain neuroscience education for pain-related disability after breast cancer surgery: study protocol for a randomized controlled trial

Lore Dams^a^, Elien Van der Gucht^b,c^, Mira Meeus^a^, Nele Devoogdt^b,c,d^, Ann Smeets^e,f^, Bart Morlion^g^

^*a*^*Department of Rehabilitation Sciences and Physiotherapy, Faculty of Medicine and Health Sciences, University of Antwerp, Antwerp, Belgium,* ^*b*^*Department of Rehabilitation Sciences, KU Leuven–University of Leuven, Leuven, Belgium,* ^*c*^*Department of Physical Medicine and Rehabilitation, University Hospitals Leuven, Leuven, Belgium,* ^*d*^*Department of Vascular Surgery, University Hospitals Leuven, Leuven, Belgium,* ^*e*^*Multidisciplinary Breast Centre, University Hospitals Leuven, Leuven, Belgium,* ^*f*^*Department of Surgical Oncology, KU Leuven–University of Leuven, Leuven, Belgium,* ^*g*^*Leuven Centre for Algology and Pain Management, University Hospital Leuven, Leuven, Belgium*

**Objectives:** To examine the effectiveness of PNE in female breast cancer patients on postoperative pain, physical-, mental- and work-related functioning.

**Methods:** A double-blinded randomized controlled trial will be conducted in the university hospitals of Leuven. One hundred eighty-four participants need to be recruited. All participants receive standard physiotherapy once or twice a week during intensive phase (4 months postoperatively) and once or twice a month during maintenance phase (4–8 months postoperatively). Additionally, participants in both the intervention and control group attend three 30-minute individual educational sessions (intervention: PNE, control: biomedical education) during intensive phase and 3 “booster sessions” during maintenance phase. The primary outcome parameter is pain-related disability 1 year after surgery. Secondary outcomes are other dimensions of pain, physical-, mental- and work-related functioning up to 18 months after surgery.

## Daily physical activity levels are predictive of the effectiveness of conditioned pain modulation

Sophie Van Oosterwijck^a^, Mira Meeus^a,b,c^, Jacob van der Wekken^b^, Linda Hermans^a,c^, Jessica Van Oosterwijck^a,b,c,d^

^*a*^*SPINE Research Unit Ghent, Department Rehabilitation Sciences, University of Ghent, Belgium,* ^*b*^*Department of Rehabilitation Sciences and Physiotherapy, University of Antwerp, Belgium,* ^*c*^*Pain in Motion international research group,* *www.paininmotion.be**,* ^*d*^*Research Foundation—Flanders (FWO), Belgium*

**Objectives:** To determine whether daily physical activity levels are predictive of conditioned pain modulation (CPM) in healthy adults.

**Methods:** Seven days prior to CPM assessment, physical activity levels of 105 healthy adults were evaluated using self-report and continuous accelerometry. CPM was evaluated through a standardized heterotopic noxious conditioning stimulation protocol. Before, during, and after immersion of the non-dominant hand into hot water, pressure pain thresholds were evaluated at the dominant hand, neck, and leg. Hierarchical regression was conducted to determine a predictive relationship between physical activity and CPM efficacy, meanwhile controlling for potential confounders. Higher self-reported levels of moderate physical activity (eg, cycling <16 km/h) significantly predicted greater CPM magnitude. When the number per steps per day, as registered with accelerometry, was ≥10.000 (=active) or ≥12.500 (=highly active) this significantly predicted more efficacious CPM. Physical activity appears essential to the efficacy of CPM, and thus forms one of the confounding factors which influence CPM. Walking and performing moderately intense activities are achievable for chronic pain patients who are known to have dysfunctional CPM, and the current results are promising for the implementation of these activities in rehabilitation programs aimed at improving CPM and combat pain.

## The role of pain cognitions in healthcare utilization in patients undergoing surgery for lumbar radiculopathy: a randomized controlled trial

Eva Huysmans^a^, Koen Putman^b^, Lisa Goudman^a^, Iris Coppieters^a^, Kelly Ickmans^a^, Ronald Buyl^c^

^*a*^*Department of Physiotherapy, Human Physiology and Anatomy, Faculty of Physical Education & Physiotherapy (KIMA), Vrije Universiteit Brussel, Brussels, Belgium,* ^*b*^*Department of Public Health (GEWE), Faculty of Medicine and Pharmacy, Vrije Universiteit Brussel, Brussels, Belgium,* ^*c*^*Department of Biostatistics and Medical Informatics, Vrije Universiteit Brussel, Brussels, Belgium*

First, to explore the relationship between pain cognitions and healthcare use (HCU) of patients scheduled for lumbar radiculopathy surgery. Second, to investigate the mediating role of pain cognitions in the mechanism behind HCU post-surgery.

**Methods:** Eligible patients (n = 120) are randomized to a perioperative pain neuroscience education (targeting pain cognitions) group or control group. HCU, Tampa Scale for Kinesiophobia (TSK), Pain Catastrophizing Scale (PCS) and Pain Vigilance and Awareness Questionnaire (PVAQ) are assessed at baseline and until 2 years post-surgery. Baseline associations are investigated univariately. Therapy effects and causal interactions are investigated multivariately. Preliminary baseline findings (n = 100) show that, patients scoring above the PCS cut-off use more types of analgesics (*P* = 0.017). High numbers of neurosurgeon visits are associated with worse catastrophizing (*P* = 0.069) and especially rumination (*P* = 0.023). Strong opioid use is also related to higher PCS rumination scores (*P* = 0.076). Using analgesics in general is related to higher PVAQ attention to pain subscale scores (*P* = 0.094).

**Conclusions:** Preliminary baseline findings underscore the possible association between pain cognitions and HCU. However, based on these explorative analyses no strong conclusions can be made. Further analyses will provide insight in the clinical relevance of these relationships and possible causal interactions between pain cognitions and HCU.

## Body perception in fibromyalgic patients: a mixed-method research study

Antonello Viceconti^a^, Deborah Luzzi^a^, Michela Barisone^b^, Alvisa Palese^c^, Marco Testa^a^

^*a*^*Department of Neuroscience, Rehabilitation, Ophthalmology, Genetics, Maternal and Child Health, University of Genova, Campus of Savona, Savona, Italy,* ^*b*^*Asl 2 Sistema Sanitario Regione Liguria,* ^*c*^*Department of Medical and Biological Sciences, University of Udine*

Preliminary observations report “phantom sensations” of swelling hands and feet in fibromyalgic patients (), similar to those described in neuropathic conditions. Patients may not refer this kind of “bizarre” perceptual disturbances, if not directly questioned, for fear of being considered mentally disturbed. Moreover, a specific test or validated questionnaires are not available thus, the only way to explore this phenomenon remain the patient's history itself.

**Methods:** A mixed-method study will be conducted on a convenience sample of 100 adult patients. A series of questionnaires will be administered to describe the clinical features of the sample. Patients reporting at least 2 affirmative answers on customized survey investigating body perception disturbances will be considered eligible for qualitative inquiry. Subjective experience of own's body perception will be explored through semi-structured interviews: answers will be audio-recorded and transcribed verbatim to perform the descriptive phenomenological analysis.

**Results:** in mixed-method research design quantitative and qualitative data collection are sequential: findings emerging will represent the integration of both datasets. A better knowledge about body perception may be a starting point to obtain prevalence data on perceptual dysfunctions in fibromyalgic patients, and to study a possible correlation between these phenomena and clinical or demographic features.

## Using a humanoid robot to distract children with cancer undergoing painful procedures: a pilot randomized controlled trial

Emma Rheel^a,b^, Kelly Ickmans^a,b,c,d^, Tine Vervoort^e^, Anneleen Malfliet^a,b,c,d^, Roselien Pas^a,b^

^*a*^*Department of Physiotherapy, Human Physiology and Anatomy, Faculty of Physical Education & Physiotherapy, Vrije Universiteit Brussel, Belgium,* ^*b*^*Pain in Motion international research group,* *www.paininmotion.be**,* ^*c*^*Department of Physical Medicine and Physiotherapy, Universitair Ziekenhuis Brussel, Belgium,* ^*d*^*Research Foundation—Flanders (FWO), Brussels, Belgium,* ^*e*^*Department of Experimental-Clinical and Health Psychology, Ghent University, Belgium*

Cancer survivors are more likely to develop chronic pain (1), which may not only arise from treatments but also from children's pain memory (2). Humanoid robot distraction has proven to be effective in reducing healthy children's pain and distress during vaccinations (3). Whether these benefits generalize to children with cancer and pain memories, needs to be examined. Children (8–12 years) with a portal catheter and their parents will be recruited. Baseline assessments include child's anticipated pain and fear, self-efficacy, attention control, attention bias, energy-balanced behavior, pressure hyperalgesia, child's and parent's catastrophizing, parental emotional and behavioral responses. After randomization to control group (usual care) or intervention group (robot distraction), child's experienced self-reported pain and fear and parent's pain catastrophizing and emotional response will be assessed. Two weeks later child's pain and fear memory and future pain expectancies will be assessed by telephone. Recalled pain and fear ratings higher than initial reports are considered indicative of negative memory biases. No results yet to display. No conclusions yet to display.

## Influence of education level on the effectiveness of pain neuroscience education: a secondary analysis of a randomized controlled trial

Thomas Bilterys^a,b,c^, Jeroen Kregel^a,c^, Jo Nijs^a,b,d^, Mira Meeus^a,c,e^, Nathalie Roussel^a^, Lieven Danneels^c^

^*a*^*Pain in Motion International Research Group,* *http://www.paininmotion.be**,* ^*b*^*Department of Physiotherapy, Human Physiology and Anatomy, Faculty of Physical Education and Physiotherapy, Vrije Universiteit Brussel, Brussels, Belgium,* ^*c*^*Department of Rehabilitation Sciences and Physiotherapy, Faculty of Medicine & Health Sciences, Ghent University, Ghent, Belgium,* ^*d*^*Department of Physical Medicine and Physiotherapy, University Hospital Brussels, Brussels, Belgium,* ^*e*^*Department of Rehabilitation Sciences and Physiotherapy (MOVANT), Faculty of Medicine and Health Sciences, University of Antwerp, Antwerp, Belgium*

**Objectives:** The effects of pain neuroscience education (PNE) at group level are rather small and little is known about personal factors (eg, level of education [LoE]) potentially influencing the effectiveness of PNE. The aim of this study is to investigate the influence of LoE on the effectiveness of pain neuroscience education in chronic spinal pain (CSP) patients.

**Methods:** A total of 120 people with nonspecific CSP (ie, neck and back pain) were randomly assigned to the control or experimental group. Every patient received 3 education sessions of PNE or neck/back school. Several self-report questionnaires were used to measure treatment outcomes (disability, catastrophizing, kinesiophobia, perceptions and vigilance). Based on both LoE and group allocation, 6 groups were formed.

**Results:** Analyses are ongoing. Differences between groups will be checked using repeated measures ANOVA and Bonferroni post-hoc analysis.

**Conclusions:** This is the first study to provide insight in the influence of LoE on the effectiveness of PNE. No follow-up measurements are available, resulting in the inability to investigate long-term effects. The results from this study may contribute to the identification of patients who benefit the most from PNE and those who are less susceptible, based on LoE.

## Relations between nutrition and chronic pain in cancer patients and cancer survivors

Sevilay Tumkaya^a,b^, Ömer Elma^a,b^, Peter Clarys^a^, Tom Deliens^a^, Jo Nijs^a,b,c^, Anneleen Malfliet^a,b,c,d,e^

^*a*^*Vrije Universiteit Brussel, Belgium,* ^*b*^*Pain in Motion, international research group,* ^*c*^*University Hospital Brussel, Belgium,* ^*d*^*Research Foundation Flanders, Belgium,* ^*e*^*Ghent University*

**Objectives:** To date, no clear overview exists on the relation between chronic pain and nutrition in cancer patients and cancer survivors. Therefore, the aim of this systematic review is to identify relevant evidence regarding this association to provide guidance for future research in this field.

**Methods:** This systematic review will be performed according to the Preferred Reporting Items for Systematic review and Meta-Analyses (PRISMA) guidelines. PubMed, Web of Science and Embase databases will be searched. Firstly, titles and abstracts of the obtained articles will be screened and then second screening will be based on full-text. The Cochrane Collaboration's tool (for randomised controlled trials) and, the Newcastle-Ottawa Scale (for observational studies) will be used for risk of bias assessment of studies. Data will be extracted using a data extraction form, according to collected data, analytical or descriptive synthesis will be performed.

**Results:** Results are not available yet, as the search is ongoing. Results will be available for presentation during the PSiM congress.

**Conclusions:** This review is important to see if nutritional interventions might be useful in pain management for cancer patients and survivors.

## Noninvasive intracranial pressure monitoring in patients with chronic migraine

Denise Martineli Rossi^a^, Sérgio Mascarenhas^b^, Débora Bevilaqua-Grossi^a^, Gabriela Ferreira Carvalho^a^, Hugo Celso Dutra Souza^a^, Antônio Carlos Santos^a^

^*a*^*Department of Healthy Science, Ribeirão Preto Medical School, University of São Paulo,* ^*b*^*São Carlos Institute of Physics, University of São Paulo*

Recent studies have suggested a possible relationship between the dysregulation of cerebrospinal fluid and intracranial pressure in the central nervous system with symptoms such as allodynia and hyperalgesia. The role of increased intracranial pressure has been investigated in patients with chronic migraine whose pathophysiology comprises a complex neurovascular dysfunction. However, this question has still not been investigated thoroughly considering the risks of the invasive methods of measuring intracranial pressure. The aim of this study is to investigate possible alterations in the waveform morphology through noninvasive intracranial pressure in patients with chronic migraine. Thirty women patients with chronic migraine and 30 healthy age matched controls will be evaluated. The noninvasive intracranial pressure monitoring will be performed by a valid method patented by Brazilian researchers that consists of an extracranial deformation sensor positioned in the patients' scalp, which will allow registration of intracranial pressure waveforms. Data will be continuously and simultaneously collected with blood pressure and heart rate measurements during 20 minutes, after 10 minutes-resting, in the supine position. Parameters obtained from the waveforms will be analyzed and compared between groups such as, P1 slope and P2/P1 index. Results and conclusions: The project is in its initial phase of pilot testing.

## Associations between health-related quality of life and nociceptive modulation and employment status in patients with lumbar radiculopathy

Wouter Van Bogaert^a^, Koen Putman^b^, Iris Coppieters^a^, Lisa Goudman^c^, Jo Nijs^a^, Maarten Moens^c^

^*a*^*Department of Physiotherapy, Human Physiology and Anatomy, Faculty of Physical Education & Physiotherapy (KIMA), Vrije Universiteit Brussel,* ^*b*^*Department of Public Health (GEWE), Faculty of Medicine and Pharmacy, Vrije Universiteit Brussel,* ^*c*^*Department of Neurosurgery, Universitair Ziekenhuis Brussel*

**Objectives:** This study primarily aims to compare health-related QOL of patients with lumbar radiculopathy to a group of healthy subjects. Secondary aims are to assess the association between the patients' health-related QOL and (1) altered endogenous nociceptive modulation and (2) their employment status and sick leave duration.

**Methods:** Health-related QOL is measured by the Short form 36 item Health Survey (SF-36). Endogenous nociceptive modulation was tested via an electrical pain stimulation testing protocol where patients' electric detection thresholds (EDT) and electric pain thresholds (EPT) were evaluated. Conditioned pain modulation paradigm and temporal summation protocol were used to assess endogenous nociceptive inhibition and wind-up. Employment status and sick leave duration were evaluated using self-reported questionnaires. Mean scores of the SF-36 scales were compared between patients and the healthy group. Within the patient group multi-variate analyses were used to find associations between the SF-36 and endogenous nociceptive modulation, employment status and sick leave duration.

**Results:** Results of the present research questions will be available at the time of the colloquium.

**Conclusions:** It is hypothesized that patients with lumbar radiculopathy will report lower values for health-related QOL. An association between lower health-related QOL and disturbed endogenous nociceptive modulation, longer sick leave duration and unemployment is expected.

## Effects of early virtual reality-based rehabilitation in patients with total knee arthroplasty: a randomized controlled trial

Silvia E. Gianola^a^, Elena Stucovitz^b^, Greta Castellini^a,c^, Mariangela Mascari^d^, Francesco Vanni^d^, Irene Tramacere^e^

^*a*^*IRCCS Istituto Ortopedico Galeazzi, Unit of Clinical Epidemiology, Milan, Italy,* ^*b*^*IRCCS Istituto Ortopedico Galeazzi, Motion Analysis Laboratory, Milan, Italy,* ^*c*^*Department of Biomedical Sciences for Health, University of Milan,* ^*d*^*IRCCS Istituto Ortopedico Galeazzi, Center for Sports Rehabilitation, Milan, Italy,* ^*e*^*Scientific Directorate, Fondazione IRCCS Istituto Neurologico Carlo Besta, Milan, Italy*

To assess the efficacy of early inpatient rehabilitation using a virtual reality-based vs traditional rehabilitation after primary total knee arthroplasty (TKA), we included subjects, aged 45 to 80 year old, receiving primary TKA who were randomly assigned to either virtual-based or traditional rehabilitation for at least 5 sessions, 60 minutes each one. The primary outcome was pain intensity. The secondary outcomes were: knee disability, health-related quality of life, global perceived effect, functional independent measure, medications use, hip isometric strength, flexion range of motion, and proprioception. Outcomes were assessed at baseline and at discharge (around 10 days after surgery). Eighty-five patients received either virtual reality-based (n = 44) or traditional (n = 41) rehabilitation; there were 11 drop-outs (9 in the virtual reality-based rehabilitation). No significant difference in pain reduction or other outcomes was found between the groups (mean difference 5.9, 95% CI −4.6 to 16.5, *P* = 0.2660), whereas a statistically significant improvement in global proprioception after virtual reality-based rehabilitation (mean difference 13.6, 95% CI 5.2–22.0, *P* = 0.0020) was noted. Virtual reality-based rehabilitation is not superior to traditional one for pain or other outcomes but it seems to improve the global proprioception in TKA patients.

## Chronic low back pain and nutrition in adults: a cross-sectional, observational study

Omer Elma, Sevilay Tumkaya Yilmaz, Peter Clarys, Tom Deliens, Jo Nijs, Anneleen Malfliet

Department of Physiotherapy, Human Physiology and Anatomy, Faculty of Physical Education & Physiotherapy, Vrije Universiteit Brussel, Brussels, Belgium

**Objectives:** It is well known that chronic low back pain is associated with many factors including mental health, lifestyle factors and cognitive factors etc. One of the lifestyle factors might be nutrition. However, this association is not clear yet. Thus, aim of this study is to investigate association between dietary intakes, and diet quality with the chronic low back pain by comparing healthy-pain free control group.

**Methods:** In this cross-sectional study there will be 2 groups; chronic low back pain (n = 64) and healthy-pain free participants (n = 64). The examination will include general health interview, dietary interview, body composition measurements and pain measurements. Nutritional data will be collected with a validated food frequency questionnaire to find out Mediterranean Diet Score, Healthy Eating Index-2015 score, specific nutrient intakes and plant-based diet score of participants. Visual analogue scale, central sensitization inventory, pressure pain threshold, temporal summation test and conditioned pain modulation test will be used as pain outcome measures. Additionally, body composition, physical activity level and quality of life will be examined. Collected data will be analysed by using SPSS program.

**Results:** This study is still ongoing.

**Conclusions:** Results of this study might give us an important insight about the association between nutrition and chronic low back pain.

## Assessment of the effects caused by mechanical stimulation on peripheral nervous system

Giacomo Carta^a^, Federica Fregnan^b^, Giovanna Gambarotta^a^, Stefano Geuna^a^

^*a*^*Department of Clinical and Biological Sciences, University of Turin, Orbassano (TO), Italy,* ^*b*^*Neuroscience Institute Cavalieri Ottolenghi (NICO), Orbassano (TO), Italy*

**Objectives:** Since 1990 low back pain and neck pain are the leading causes of disability worldwide. In the last decades it has been shown that the selective repeated tension of the Peripheral Nervous System (PNS), also known as neurodynamic treatment (NDT) can be successful in pain modulation of patients affected by chronic and acute back and neck pain. Nowadays the biological effects involved in NDT still unknown and no standard protocol is available. The study aim to assess the effects of NDT on PNS cells in order to develop a protocol of treatment for animal models of acute and chronic pain.

**Methods:** immortalized cell lines of motor and sensitive neurons (NSC34 and 50B11) were used. A repeated 4 arms randomized controlled trial was performed for each immortalized cell line. Cells were seeded on pre-coated silicone membranes and repeated tension protocols were administered using a bioreactor developed and used ad hoc. Morphological, Genetic and protein expression analysis were performed.

**Results:** A standardized protocol of NDT was possible to be defined. Preliminary results have shown that NDT seems to have no side effects and can affect neurites orientation, cell differentiation and avoids apoptosis. Interestingly, a protocol of NDT downregulates the expression of TLR2, a gene linked to mechanical allodynia and also up regulates genes involved in neurites growth.

**Conclusions:** Results from our preliminary experiments are promising and they suggest that NDT can be standardized being immediately translatable in clinical practice and promote the regeneration processes in motor and sensory neurons.

## Pain neuroscience education for children with functional abdominal pain related disorders: a randomised controlled pilot study

Roselien Pas^a^, Sophie Van Oosterwijck^b^, Mira Meeus^b^, Laurence Leysen^c^, Emma Rheel^c^, Ann Roete^d^

^*a*^*Department of Physiotherapy, Human Physiology and Anatomy, Faculty of Physical Education and Physiotherapy, Vrije Universiteit Brussel, Brussels, Belgium,* ^*b*^*Department of Rehabilitation Sciences and Physiotherapy, Faculty of Medicine and Health Sciences, Ghent University, Belgium,* ^*c*^*Vrije Universiteit Brussel (VUB), Department of Physiotherapy, Human Physiology and Anatomy (KIMA), Faculty of Physical Education & Physiotherapy, Brussels, Belgium,* ^*d*^*University Hospital Antwerp, Antwerp, Belgium*

**Objectives:** This study explores the effectiveness and feasibility of a PNE program for children with functional abdominal pain-related disorders (FAPD).

**Methods:** Children aged between 6 and 12 years and diagnosed with FAPD were randomly assigned to either the experimental or control group, both receiving 2 treatment sessions with a 3-week time interval between the sessions. The experimental group received; (1) usual care including bio-medical directed education about the gastro-intestinal system and breathing exercises and (2) a PNE session (PNE4Kids). Similar to adult PNE, PNE4kids included the explanation about the cause of pain, a brief summary of relevant pain mechanisms and the integral role of psychosocial and physical factors in maintaining pain. The control group received 2 usual care sessions. Pressure algometry and conditioned pain modulation were assessed at baseline and 3 weeks' follow-up. The child's pain intensity, functional disability, pain-related fear, as well as parental pain catastrophizing were assessed at baseline, after each treatment session and 3 weeks' follow-up.

**Results:** Intervention and feasibility outcomes concerning the study design and procedures are pending and will be presented at the congress.

**Discussion:** Data resulting from this explorative study will lay the foundation for larger randomized trials in the future.

## Pain-related sensory and psychological factors may contribute to walking impairment in adults with low back pain

Katie A. Butera^a^, Steven Z. George^b^, Paul Freeborn^c^, Mark D. Bishop^a^, Stephen A. Coombes^d^, Emily J. Fox^a^

^*a*^*University of Florida, Department of Physical Therapy,* ^*b*^*Duke University, Duke Clinical Research Institute,* ^*c*^*Brooks Rehabilitation; Motion Analysis Center,* ^*d*^*University of Florida, Department of Applied Physiology and Kinesiology*

**Objectives:** There is a poor understanding of LBP functional performance and studies rarely consider how nervous system factors impact function. The purpose of this study was to determine whether nervous system factors are associated with walking function in adults with LBP.

**Methods:** Thirty-seven adults with LBP (18 males; mean age = 45) underwent pain sensitivity testing and completed psychological questionnaires. Participants rated pain during walking and completed the Oswestry Disability Index. Walking speed was measured during a “fastest-comfortable” and “obstacle negotiation” condition. Data were separated into fast and slow groups. One-way ANOVAs tested group differences on pain sensitivity, pain during movement, psychological constructs, and disability.

**Results:** Groups reported low disability (11%–23%). Slow groups were 0.6 m/s slower than fast groups for both conditions (*P* < 0.05). The “fastest comfortable” slow group demonstrated higher pain sensitivity, lower positive coping, higher pain during walking, and higher disability (*P* < 0.05). Similarly, the “obstacle negotiation” slow group demonstrated higher pain sensitivity, higher catastrophizing, lower positive coping, higher pain during walking, and higher disability (*P* < 0.05).

**Conclusions:** Despite low self-reported disability, nervous system factors representing different constructs were associated with walking speed during 2 conditions. Factor clusters may be important for characterizing LBP clinical phenotypes and guiding development of personalized interventions.

## Spinal cord injury and emotional responses: a topography in empathy for pain

Giulia Caffini^a^, Michele Scandola^b^, Valentina Moro^b^

^*a*^*Physiotherapist at Fisiopoint Italia srl,* ^*b*^*NPsy-Lab VR, Department of Human Science, University of Verona*

**Objectives:** Spinal Cord Injury (SCI) determines changes in cognitive functions linked to the body and motion,^1^ associated to the sensorimotor side of Pain.^2,3^ This study wants to analyse empathy for pain (EfP) in SCI in order to consider also the affective and emotional aspect. This by investigating if: (1) if the sensorimotor functions in Complete Paraplegics can influence EfP; (2) if the affective emotional component of pain has a somatotopographic organization too; (3) implicit responses, compared to explicit ones.

**Material and methods:** An experimental study conducted on 49 subjects participated: 21 excluded, 28 included (n = 14 experimental group—Complete Paraplegics; n = 14 control group—Healthy). The stimuli: videos in virtual reality characterised by syringe pic or Q-tip touch, hand or foot or neutral target, perspective in first-person (1 PP) or third-person (3 PP). The responses: GSR (Galvanic Skin Response), EMG of the Corrugator and Zygomaticus, and NRS questionnaire.

**Results:** Significant interactions founded between: (1) group and condition foot-syringe [z(GRS), *P* = 0.016] for GSR; 1 PP and conditions foot-syringe, hand-syringe [(zCor-zZig), *P* <0.01] for EMG. Data confirmed by subjects' explicit answers regarded ownership. There is: (1) a somatotopographic organization of EfP, linked to afferent and motor efferent signals; (2) an influence between the emotional component of Pain and sensorimotor functions.

## Rehabilitation intervention in randomized controlled trials for low back pain: are they statistically significant and clinically relevant?

Greta Castellini^a,b^, Silvia Gianola^a^, Davide Corbetta^c,d^, Lorenzo Moja^b,e^

^*a*^*IRCCS Galeazzi Orthopaedic Institute, Unit of Clinical Epidemiology, Milan, Italy,* ^*b*^*Department of Biomedical Sciences for Health, University of Milan,* ^*c*^*IRCCS San Raffaele Hospital, Rehabilitation and Functional Recovery Department, Milan,* ^*d*^*Vita-Salute San Raffaele University, Physiotherapy Degree Course, Milan,* ^*e*^*Clinical Epidemiology Unit, I.R.C.C.S. Orthopaedic Institute Galeazzi, Milan, Italy*

**Objectives:** To assess if treatment effects of randomized controlled trials (RCTs) for low back pain (LBP) are statistically significant and clinically relevant, and if RCTs were powered to achieve clinically relevant differences on continuous outcomes.

**Methods:** RCTs included in Cochrane Systematic Reviews on LBP rehabilitation and having sample size calculation and a planned minimal important difference were considered. We calculated the proportion of RCTs classified as “statistically significant and clinically relevant,” “statistically significant but not clinically relevant,” “not statistically significant but clinically relevant,” and “not statistically significant and not clinically relevant.” We investigated how many times the mismatch between statistical significance and clinical relevance was due to inadequate power.

**Results:** We identified 42 RCTs encompassing 81 intervention comparisons. Sixty percentage (25 RCTs) were statistically significant while only 36% (15 RCTs) were both statistically and clinically significant. Most trials (38%) did not discuss the clinical relevance of treatment effects when results did not reached statistical significance. Among trials with non-statistically significant findings, 60% did not reach the planned sample size.

**Conclusions:** Scarce diligence or frank omissions of important tactic elements of RCTs, such as clinical relevance, and power, decrease the reliability of study findings to current practice.

## Epigenetics of BDNF and its relationship with central sensitisation in patients with chronic widespread pain and chronic fatigue syndrome

Andrea Polli^a^, Lode Godderis^b^, Kelly Ickmans^a^, Jo Nijs^a^

^*a*^*Pain in Motion (PiM) research group, Department of Physiotherapy, Human Physiology and Anatomy, Vrije Universiteit Brussel,* ^*b*^*Department of Public Health and Primary Care, KU Leuven*

**Introduction:** Sensitivity of the central nervous system is a relevant feature in Chronic widespread pain (CWP). BDNF is a neurotrophins that increase central sensitization (CS). We aimed to explore the relation between BDNF and CS and its possible role in CWP, as well as investigate epigenetic mechanisms implicated in BDNF protein expression.

**Methods:** Fifty-four women (28 with CWP; 26 healthy subjects, HSs) were enrolled. Participants were assessed twice within 5 days measuring pain, CS, pain sensitivity, BDNF protein levels, and DNA methylation of BDNF gene.

**Results:** CS inventory was strongly associated to pain thresholds in both assessments. ICCs showed good stability of pain thresholds and BDNF levels (Cronbach's a > 0.8). RM ANOVA, controlling for time and variability of measures, showed BDNF to be higher in people with CFS (F = 11.013, *P* = 0.002; 17.23 [4.45] ng/mL vs 14.03 [3.89] ng/mL). BDNF levels correlated with CS inventory and pain thresholds. We found no between-group differences in DNA methylation of BDNF gene nor significant correlation between BDNF expression and BDNF gene methylation.

**Conclusions:** BDNF protein concentration is stably higher in patients with CFS and it is associated with CS. Epigenetic mechanisms investigated in this research were not able to explain the difference in BDNF expression.

Meeus M, Nijs J. *Clin Rheumatol* 2007;26:465–473. Nijs J, et al. *Expert Opin Ther Targets* 2015;19:565–576. Polli A, et al. *Arch Phys Med Rehabil* In press (2018).

## Measuring therapeutic alliance in multidisciplinary pain rehabilitation

Davy Paap^a^, Grieteke Pool^b^, H. Rita Schiphorst Preuper^a^, Michiel F. Reneman^a^, Jan H.B. Geertzen^a^, Pieter U. Dijkstra^a^

^*a*^*Department of Rehabilitation Medicine, University Medical Center Groningen, University of Groningen, Groningen, The Netherlands,* ^*b*^*Section Health Psychology, University Medical Centre Groningen, University of Groningen, Groningen, The Netherlands*

To acquire a better understanding how patients with persistent pain, perceive their therapeutic alliance with a team of rehabilitation professionals, to provide a theoretical model regarding the therapeutic alliance in a multidisciplinary rehabilitation setting.

**Methods:** Twenty-one patients with persistent pain were semi-structured interviewed. Prior to the interview the Working Alliance Inventory-Rehabilitation Dutch Version, Adults Attachment Style Questionnaire and the Interpersonal Circumplex Questionnaire were filled in by patients. Patients were asked to reflect to the different professionals, the treatment provided, comfort with negative feeling and negotiable stance, and their own role in the treatment process. The study was performed according to Grounded Theory. The questionnaires were used for within patient's analyses. At the first glance patients experienced a strong therapeutic alliance, however deeper reflection gave more nuances, and relation ruptures came to light. The attachment style of the patient may have influenced the strength of the therapeutic alliance. Avoiding of confrontation in treatment occurred consciously and unconsciously, and lead to ruptures in the therapeutic alliance.

**Conclusions:** The context and the systems of patients and the healthcare professionals is important for strength of the therapeutic alliance, it seems that these aspects are not measured within the existing therapeutic alliance measurements.

## Barriers and facilitators with regards to the usability of a blended intervention in patients with medically unexplained physical symptoms

S. Toonders^a^, P.E. van Westrienen^a^, M. Nieboer^b^, E.J.M. Wouters^b^, C. Veenhof^c^, M.F. Pisters^a^

^*a*^*Center for Physical Therapy Research and Innovation in Primary Care, Leidsche Rijn Julius Health Care Centers, Utrecht, The Netherlands,* ^*b*^*Institute of Allied Health Professions, Fontys University of Applied Sciences, Eindhoven, The Netherlands,* ^*c*^*Physical Therapy Research, department of Rehabilitation, Nursing Science and Sport, Brain Center Rudolf Magnus, University Medical Center Utrecht, Utrecht, The Netherlands*

**Objectives:** To determine barriers and facilitators with regards to the usability of a proactive, blended and multidisciplinary intervention in patients with medically unexplained physical symptoms from the patient's perspective.

**Methods:** Semi-structured interviews were held with patients that completed the entire PARASOL intervention program. Using the system usability scale, both patients with high and low satisfaction were included. The topic list served as a guide in the interviews, and was compiled based on a conceptual framework for evaluation of eHealth and determinants of implementation of health care innovations.

**Results:** A total of 13 patients were interviewed after which saturation was achieved. Five patients with low user satisfaction, 4 patients with average and 4 patients with high user satisfaction were interviewed. The following themes emerged from the analysis of the interviews: expectations, goals and motivation, usability of online platform, patient involvement, involvement from disciplines, added value treatment.

**Conclusions:** Usability was moderate. Several themes were identified to improve the intervention. Based on identified factors, the intervention can be further improved. Furthermore, the study can offer valuable insights for future proactive and preventive blended health care programs.

## Do contemporary measures of physical function enhance the prediction of ongoing pain and disability following a whiplash trauma: protocol for a prospective observational study

Ahmed Alalawi, Nicola Heneghan, Alison Rushton, David Evans, Deborah Falla

Centre of Precision Rehabilitation for Spinal Pain (CPR Spine), School of Sport, Exercise and Rehabilitation Sciences, University of Birmingham, United Kingdom

The objective of the study is to identify individuals at risk of developing persistent pain and disability following acute whiplash trauma by combing contemporary measures of physical function together with established psychological and pain-related predictive factors. A prospective observational study will recruit 150 consecutive patients experiencing whiplash-related symptoms, admitted to a Major Trauma Centre in the United Kingdom within 7 days of their injury. Participants will be followed longitudinally for 12 months. The absolute risk of poor outcome will be measured using the Neck Disability Index measured at 6 months (scores ≥30%). Candidate predictors include patient characteristics, pain extent extracted from electronic pain drawings, and self-reported questionnaires of psychosocial factors. Furthermore, contemporary physical factors will be evaluated including the smoothness of neck movement, force steadiness, and neck muscle co-activations. Candidate predictors will be collected at baseline, 3, 6, and 12 months. In addition, fortnightly data collection of pain intensity, fear of movement, and performance on active movement tasks will be requested remotely through a developed smartphone app to monitor patient recovery trajectories. Logistic regression analysis will be performed to identify factors that are associated with poor outcomes on NDI. Not applicable.

## Modulation of the time of action of a placebo analgesic cream in healthy subjects

Eleonora Maria Camerone^a^, Alessandro Piedimonte^b^, Fabrizio Benedetti^b^, Marco Testa^a^, Elisa Carlino^b^

^*a*^*University of Genoa,* ^*b*^*University of Turin*

The present study aims to investigate whether manipulation of temporal information associated with the given treatment (placebo cream) modulates the onset of action of a placebo analgesic cream. A total of 80 subjects will be enrolled. Somatosensory stimulator with 300 ms duration and 5 Hz frequency will be used to induce a stable moderate pain sensation in the low back area. Placebo cream will be applied and different efficacy times information will be given to each group. Specifically, that the cream will be effective immediately (anticipated expectation group), after 5 minutes (matched expectation group) and after 10 minutes (postponed expectation group). In all groups, intensity of stimulation will be gradually reduced after 5 minutes from cream application. Expected Results: The onset of placebo cream analgesia is expected to vary accordingly with the given temporal suggestions. Therefore, pain ratings are expected to decrease immediately after cream application in the anticipated expectation group, after 5 minutes in the matched expectation group, and at a slower rate, compared to the matched group, in the postponed expectation group. If results are in line with what we expect temporal suggestions could be used to maximise placebo analgesia efficacy, and to enhance drugs' effectiveness, anticipating their onset.

## Who is more prone to experimentally-induced central sensitization amongst subjects with temporomandibular disorders and healthy individuals?

Timothée Cayrol^a^, Laurent Pitance^a^, Nathalie Roussel^b^, Emanuel van den Broeke^c^, André Mouraux^c^

^*a*^*Institute of Experimental and Clinical Research (IREC), Universite ´ Catholique de Louvain, Brussels, Belgium,* ^*b*^*Department of Rehabilitation Sciences and Physio-therapy (MOVANT), Faculty of Medicine and Health Sciences, University of Antwerp, Wilrijk, Belgium,* ^*c*^*Institute of Neuroscience (IoNS), Universite ´ catholique de Louvain, Brussels, Belgium*

Central sensitization (CS) is increasingly understood as a main contributor to temporomandibular disorders (TMD). Furthermore, a predisposition to develop more CS in response to nociceptive input may play a role in the maintenance of pain. The aim of this study is to assess the response of chronic TMD subjects and healthy individuals to high frequency stimulation (HFS). HFS is an experimental procedure inducing CS manifesting itself by secondary hyperalgesia. HFS will be used to induce CS in subjects with chronic TMD pain and healthy controls. The spatial extent and the duration of secondary hyperalgesia will be measured. Pain ratings, spontaneous pain and allodynia in the area of secondary hyperalgesia will also be assessed. Correlations between the response to HFS and a range of secondary outcomes will be assessed in subjects with TMD, including pain distribution, conditioned pain modulation, pressure pain thresholds and physical symptoms, functional limitations, and psychological factors. It is expected that some individuals in both groups will develop more CS in response to nociceptive input. Characteristics of secondary hyperalgesia may be correlated with the secondary outcomes in subjects with TMD.

## Psychological flexibility and beliefs about illness in patients with fibromyalgia: are there differences among patients from Latin America and Spain?

Rubén Uclés-Juárez^a^

Departament of Psychology, University of Almeria, Spain

The aim of this study is to explore the beliefs about causes, symptoms and treatment among fibromyalgia patients from Latin America and Spain. A survey was conducted among FM patients from Latin American countries and Spain. The sampling were carried out in fibromyalgia or chronic pain associations or groups from Spain and Latin American countries approached by mailing, telephone or social networks. Patients without a FM diagnostic were excluded. The survey contained questions on socio-demographic factors (age, gender, nationality, marital status, educational level, socioeconomical level, years with the illness and other illnesses); a list of symptoms related with his/her FM based on the Illness Perception Questionnaire- Revised (IPQ-R) Spanish version; the Acceptance and Action Questionnaire-II (AAQ-II) Spanish version, which measures the psychological flexibility; and 3 open-ended questions regarding fibromyalgia (believed causes of his/her illness, the most relieving pharmacological and non-pharmacological treatments for him/her). The survey was developed in Spanish. The data of the open-ended questions will be codified into agreed categories by several researchers. Moreover, descriptive analyses and comparations using Chi-square and Student's *t* tests will be performed comparing the variables among regions. A total of 243 participants responded to the survey from Latin America (N = 124), and Spain (N = 117). Data collection is ongoing. The possible differences among regions will be discussed taken into account the limitations of the study in the sampling and sample size.

## Pain education for patients with low back pain in Nepal: results from PEN-LBP feasibility clinical trial

Saurab Sharma^a^, Mark Jensen^b^, G. Lorimer Moseley^c^, J. Haxby Abbott^a^

^*a*^*Department of Surgical Sciences, Dunedin School of Medicine, University of Otago, Dunedin, New Zealand,* ^*b*^*Department of Rehabilitation Medicine, University of Washington, Seattle, WA,* ^*c*^*Sansom Institute for Health Research, University of South Australia, Adelaide, Australia*

The aims of the study were to cross-culturally adapt an evidence-based pain education (PE) package into Nepali, and assess the feasibility of conducting a randomized clinical trial (RCT) to evaluate its effectiveness in patients with low back pain (LBP) in Kathmandu, Nepal. We developed PE content in Nepali using Nepalese patients' pain stories and metaphors to deliver key pain concepts as recommended by Moseley and Butler. The content was pretested on 6 patients with LBP, and proofread by 3 Nepalese. We then conducted a two-arm, assessor-blinded, feasibility RCT in 40 individuals with non-specific LBP of any duration from Nepal. We randomized participants to either 1 hour each of PE or a guideline-based physiotherapy control group. The primary feasibility outcome measures were related to recruitment, assessor blinding, contamination, and treatment. The Nepalese PE package was comprehensible. Forty out of 70 participants approached met inclusion criteria and all consented to participate. Assessor blinding was feasible. There were no contamination between groups. All the study participants accepted PE as a treatment of LBP. We conclude that PE is an acceptable intervention for LBP in Nepal, and that conducting a RCT to evaluate effectiveness of PE is feasible in Nepal.

## Parallel vs sequential conditioned pain modulation testing using various parameters; does it make a difference?

Roland R. Reezigt^a,b^, Michel W. Coppieters^a^, Sjoerd C. Kielstra^a^, Wendy G.M. Scholten-Peeters^a^

^*a*^*Musculoskeletal Physiotherapy Sciences, Department of Human Movement Sciences, Faculty of Behavioural and Movement Sciences, Vrije Universiteit Amsterdam,* ^*b*^*Hanzehogeschool, The Academy of Health, Groningen*

The primary aim is to assess the differences between a parallel and sequential application on the magnitude of the CPM effect. Secondary, we assessed whether the intensity of the conditioning stimulus and a possible interaction with a second test stimulus influenced the CPM effect Healthy participants (n = 89) were tested in a cross sectional study consisting of 3 different study parts. Using Pressure Pain Thresholds and Heat Test Stimuli, parallel and sequential to a cold pressor test, the CPM effect was measured on 3 locations. In the first part, intensity of the cold pressor test was set at VAS 40/100. In the second part (n = 31), the intensity was set at VAS 60/100. In the third part (n = 29), with equal intensity, only pressure was used. The CPM magnitude was compared using a mixed method model, with parallel vs sequential as within and the 3 different study parts as between variable (2 × 3 model for PPT and 2 × 2 for HTS). No differences were found in CPM magnitude between the parallel and sequential protocols, nor between the 3 study parts. No preferred protocol can be recommended.

## Cognitive behavioral therapy for insomnia within a comprehensive treatment approach for chronic spinal pain: a randomized controlled trial

Eveline Van Looveren^a,b^, Thomas Bilterys^a,c^, Anneleen Malfliet^a,b,c,d,e^, Jo Nijs^a,b,e^, Kelly Ickmans^a,b,e^, Lieven Danneels^a^

^*a*^*Department of Rehabilitation Sciences and Physiotherapy, Ghent University, Ghent, Belgium,* ^*b*^*Pain in Motion International Research Group,* ^*c*^*Department of Physiotherapy, Human Physiology and Anatomy, Vrije Universiteit Brussel, Brussels, Belgium,* ^*d*^*Research Foundation-Flanders (FWO), Brussels, Belgium,* ^*e*^*Department of Physical Medicine and Physiotherapy, University Hospital Brussels, Brussels, Belgium*

**Introduction:** The major share of people suffering from chronic spinal pain (CSP) report comorbid insomnia. This study aims to examine if cognitive behavioral therapy for insomnia (CBT-I) combined with the modern neuroscience approach is effective in improving pain, sleep, physical activity and function in people with CSP and comorbid insomnia.

**Methods:** One hundred twenty participants with CSP and comorbid insomnia will be included in this multi-center randomized controlled trial. Participant will undergo a total of 18 therapy sessions in both the experimental and control group. Both groups will receive 3 sessions of pain neuroscience education, followed by alternating 6 sessions of CBT-I and 9 session of cognition-targeted exercise therapy in the experimental group and 15 sessions of cognition-targeted exercise therapy in the control group. Pain-related and sleep-related outcomes, physical activity and function will be assessed.

**Results:** Data will be collected until May 2021.

**Conclusions:** No conclusions yet.

## The use of symptomatic medication is associated with the degree of sensitization in patients with tension type headache

Matteo Castaldo^a^, Cesar Fernández-de-las-Peñas^b^, Maria Palacios-Ceña^b^, Paola Torelli^c^, Paolo Pillastrini^d^, Antonella Catena^e^

^*a*^*Center for Neuroplasticity and Pain (CNAP), SMI, Department of Health Science and Technology, Faculty of Medicine, Aalborg University, Aalborg, Denmark,* ^*b*^*Departamento de Fisioterapia, Terapia Ocupacional, Rehabilitación y Medicina Física. Universidad Rey Juan Carlos, Alcorcón, Madrid, Spain,* ^*c*^*Department of Medicine and Surgery, Headache Center, University of Parma, Italy,* ^*d*^*Department of Biomedical and Neurological Sciences, University of Bologna, Italy,* ^*e*^*Poliambulatorio Fisiocenter, Collecchio (Parma)*

Tension-type headache (TTH) is a common headache disorder, with no specific medication for its management. Our aim was to investigate the differences in clinical features and widespread pressure pain sensitivity according to the use of symptomatic medication in TTH. Individuals with TTH participated: a 1-month headache diary was used to collect clinical data and use of symptomatic medication. Pressure pain thresholds (PPTs) were assessed over the temporalis muscle, C5-C6 zygapophyseal joint, second metacarpal, and tibialis anterior muscle. One hundred and sixty eight participated, and 136 reported use of symptomatic medication; 58 took the medication at the beginning whereas 78 took the medication when the headache intensity was intense. No differences in clinical features and widespread pressure pain sensitivity was observed depending on taking or not the medication (all, *P* > 0.157). However, patients taking the symptomatic medication when the headache was intense exhibited widespread lower PPTs than those taking the medication at the beginning of the attack (all, *P* < 0.05). Conclusions: This study found that the use of symptomatic medication intake was not related to clinical and widespread pressure pain sensitivity in TTH, but it seems that consuming symptomatic medication at the beginning of the headache could be related to lower widespread pressure pain sensitivity.

## Which factors influences the central sensitisation inventory in patients the total hip replacement waiting list? Preliminary results

Luana de Bastos Satriano^a^, Leandro Fukusawa^a^, Tamiris Barbosa de Melo^a^, Nayra Rabelo Menezes^b^, Claudio Cazarini Junior^a^, Giancarlo Poleselo^a^

^*a*^*Faculdade de Ciências Médicas Santa Casa de São Paulo,* ^*b*^*Universidade Nove de Julho*

Evaluate which factors are associated with central sensitisation in patients in the first Hip Replacement waiting list.

**Methods:** A cross-sectional study that evaluate patients in Hip Replacement Waiting List. In our evaluation, the patient filled a form with demographic characteristics, personal history of the disease, pain intensity (Visual Analogic Scale [VAS]), catastrophizing (Pain Catastrophizing Scale [PCS]), function (Harris Hip Score [HHS]) and CS (CS Inventory [CSI]). We performed a logistic regression (LR) which was categorised in group 1: CSI <36 and group 2: CSI >36 and used this variable as dependent in the LR analysis.

**Results:** In a period of 4 months we evaluate 70 patients in HRW. The function by HHS showed poor score. In the CSI, 54.3% of patients presented a score of CSI <36 and 44.3% show a CSI >36. In the univariate LR 9 of 16 variables showed *P* < 0.2. The results of multivariate LR the Number of MSK complaints (OR: 4.3 CI 95%: 1.2–14.6) and the work disability (OR: 12.8; CI 95% 2.1–78.2) show statistically significant.

**Conclusions:** In conclusion Other MSK Complaints and work disability was associated with high CSI score.

## Reliability, validity, responsiveness and minimal clinically important change of the Dutch Version of the Pain Disability Index (DV-PDI) in female breast cancer patients

Elien Van der Gucht^a^, Lore Dams^b^, An De Groef^a,b^, Nele Devoogdt^a,c^, Koen Bernar^d^, Lode Godderis^e,f^, Bart Morlion^g,h^, Ann Smeets^i^, Mira Meeus^b,j^

^*a*^*Department of Rehabilitation Sciences, KU Leuven—University of Leuven, Leuven, Belgium*, ^*b*^*Department of Rehabilitation Sciences and Physiotherapy, Faculty of Medicine and Health Sciences, University of Antwerp, Antwerp, Belgium*, ^*c*^*Department of Vascular Surgery, University Hospitals Leuven, Leuven, Belgium*, ^*d*^*Department of Physical Medicine and Rehabilitation Sciences, University Hospitals Leuven, Leuven, Belgium,* ^*e*^*Centre for Environment and Health of KU Leuven, Leuven, Belgium,* ^*f*^*IDEWE, External Service for Prevention and Protection at Work, Leuven, Belgium,* ^*g*^*The Leuven Centre for Algology and Pain Management, University Hospitals Leuven, Leuven, Belgium,* ^*h*^Department of Cardiovascular Sciences, Section Anaesthesiology and Algology, KULeuven—University of Leuven, Leuven, Belgium, ^*i*^*Department of Surgical Oncology, University Hospitals Leuven, Leuven, Belgium,* ^*j*^*Pain in Motion research group, www.paininmotion.be*

**Objectives:** To assess reliability, validity, responsiveness and minimal clinically important change of the Dutch version of the Pain Disability Index. Both a cross-sectional and a prospective cohort study will be conducted within the University Hospitals of Leuven. In order to assess reliability, test-retest reliability and measurement error will be determined. Therefore, 30 female breast cancer patients will fill out the PDI twice with a 24-hour interval. Validity will be examined through both construct and known-groups validity. Construct validity is deemed sufficient if a minimum of 75% of the hypothesized correlations is met and known-groups validity will be achieved if the PDI can successfully discriminate between participants with and without pain. Responsiveness and minimal clinically important change will be assessed through an anchor-based approach over a four-month period. For this, we will follow a cohort of 30 participants, both at baseline (1 week after surgery) and 4 months thereafter. At these time points, the women will fill out the PDI and a self-reported global perceived effect scale. Responsiveness will be considered sufficient when the area under the receiver operating characteristic curve exceeds 0.70. In order to determine the minimal clinically important change, we will calculate the optimal cut off point and its sensitivity and specificity.

